# Supplementary figures and images for: Chromosome separation during Drosophila male meiosis I requires separase-mediated cleavage of the homolog conjunction protein UNO
Source: PLoS Genet. 2020 Oct 1;16(10):e1008928. doi: 10.1371/journal.pgen.1008928 (PMC7529252; doi:10.1371/journal.pgen.1008928)

## A MNM

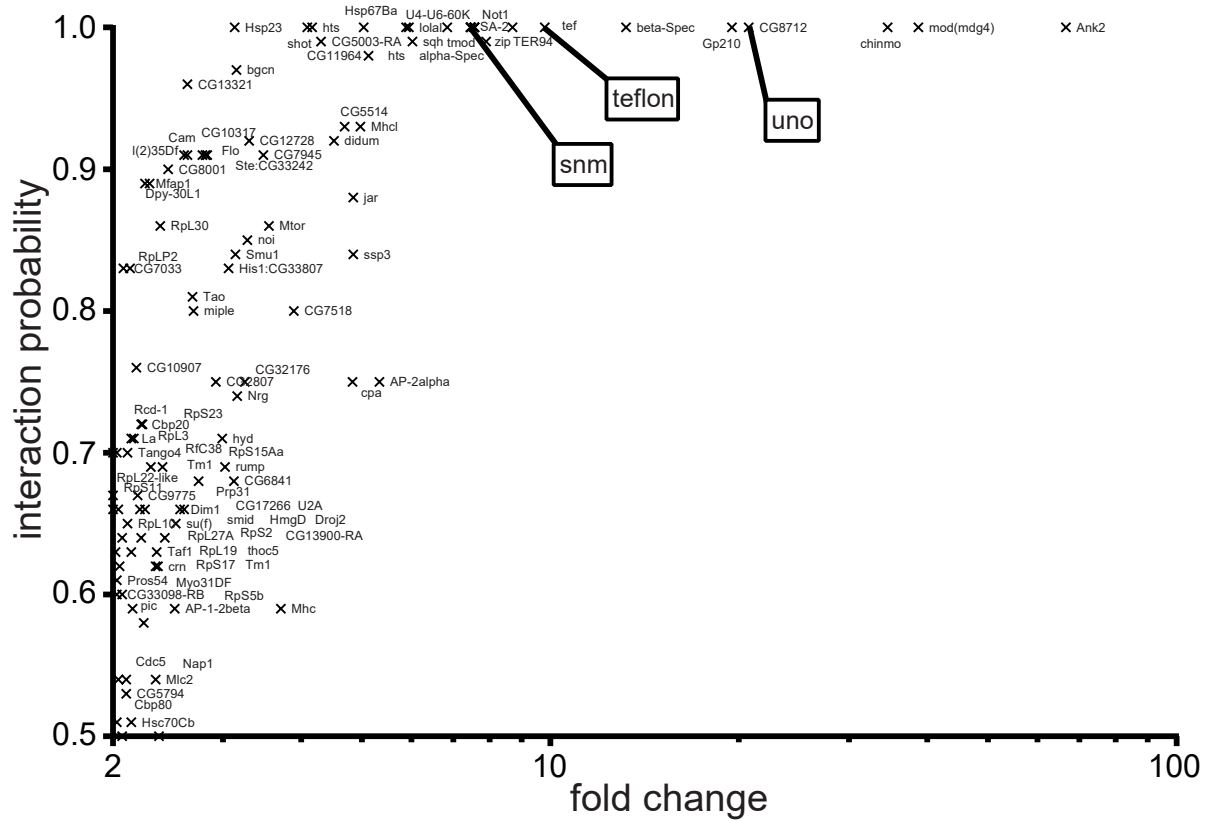

## B SNM

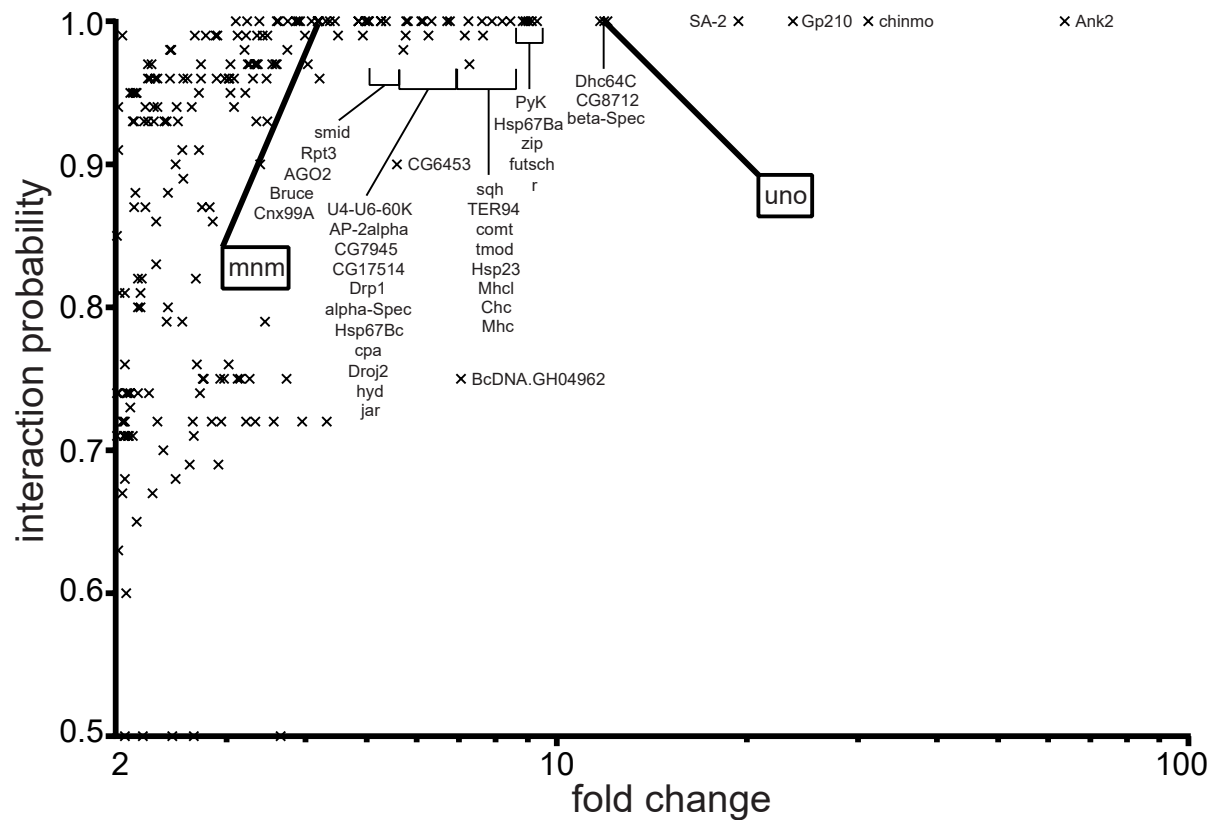

Supplement: S1 Fig — (A, B) Scatter plots display the fold change of spectral counts (in comparison to control) as well as the probability of interaction for proteins co-purified with the baits. MNM-EGFP and SNM-EGFP were used as bait proteins, EGFP as control. Mass spectrometry was used for the identification of proteins co-purified by affinity purification from testis extracts with anti-GFP. Interaction probability was calculated by the SAINTexpress algorithm. (A) MNM-EGFP interactome. (B) SNM-EGFP interactome. The values obtained for the known AHC proteins and for UNO are highlighted (boxes). To avoid overcrowding of the SNM interactome plot, only preys with a fold change higher than five were annotated. (PDF) [file pgen.1008928.s001.pdf]

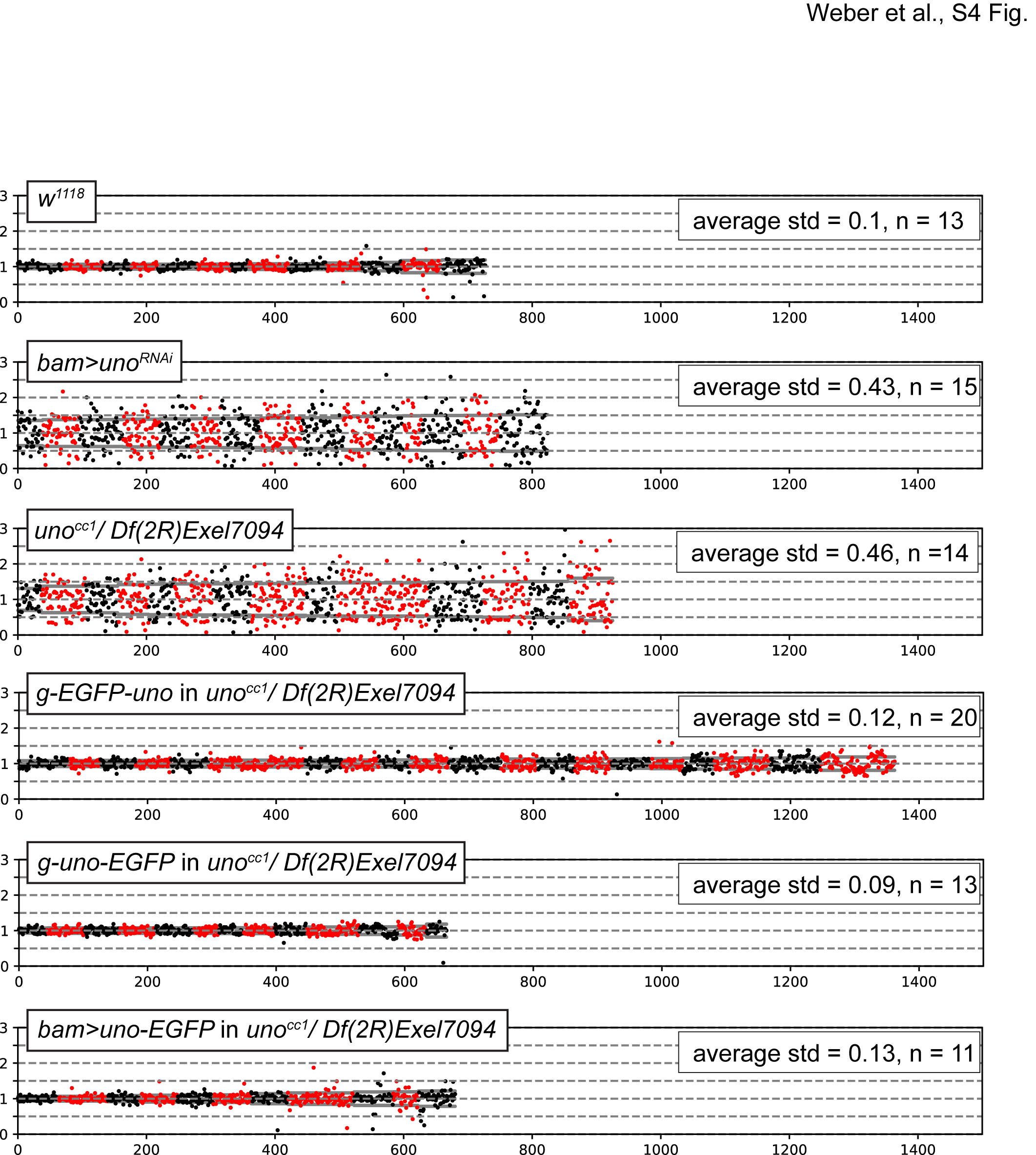

Supplement: S4 Fig — Squash preparations of testes from the indicated genotypes were labeled with a DNA stain, and cysts with early postmeiotic spermatids were imaged. DNA signal intensity in each spermatid nucleus was quantified. The average of all the individual nuclear DNA content values obtained for a given cyst was used for normalization of these values. The normalized values obtained for the nuclei within a given cyst are plotted in the same color with different cysts alternating between black and red from left to right. The standard deviation of the nuclear DNA content values within a given cyst was used for ordering the different cysts of the same genotype, with standard deviations increasing from left to right. The average standard deviation for all analyzed cysts of the same genotype is displayed along with the number of analyzed cysts. (TIF) [file pgen.1008928.s004.tif]

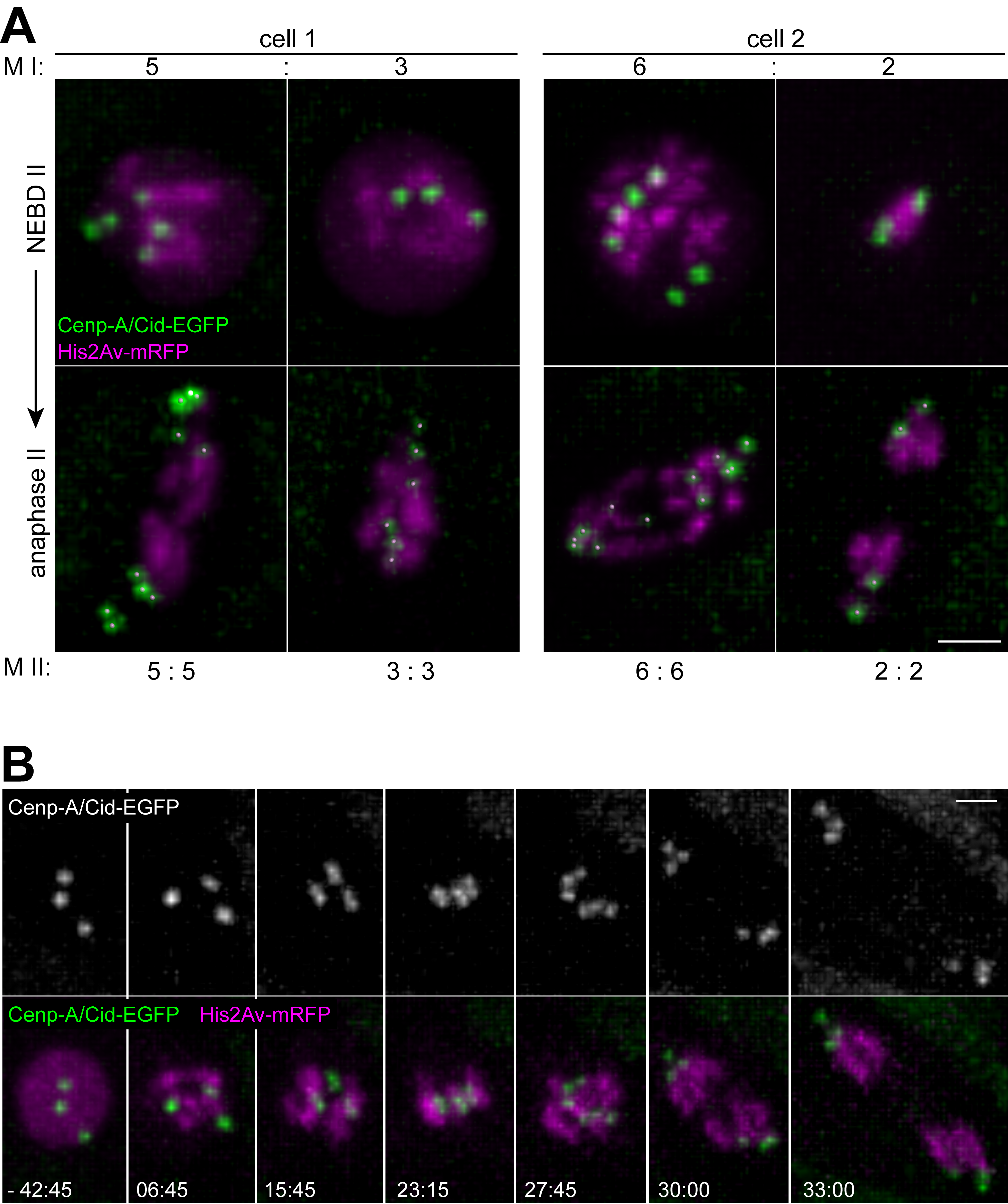

Supplement: S5 Fig — (A,B) Chromosome segregation during meiosis was analyzed by time-lapse imaging with uno null mutant spermatocytes (unocc1/ Df(2R)Exel7094) expressing Cenp-A/Cid-EGFP and His2Av-mRFP. (A) Because of random segregation of eight univalents during M I, pairs of secondary daughter spermatocytes can arise in uno null mutants with a centromere distribution that is not 4:4, as after regular separation of four bivalents during normal M I. In case of the two uno null mutant spermatocytes documented, centromeres were segregated 5:3 (cell 1) and 6:2 (cell 2) during M I. The corresponding numbers of Cenp-A/Cid-EGFP dots are clearly detectable in the still frames selected at the start of M II (NEBD II). After random segregation during M I, however, uno null mutants segregate sister centromeres regularly during M II, as illustrated with still frames selected from anaphase II with centromeres marked by small spheres. Note that spinning disc confocal microscopy of Cenp-A/Cid-EGFP cannot resolve the two sister centromeres before bi-orientation [56]. (B) Progression of a uno null mutant secondary spermatocyte through M II. The indicated times (min:sec) are given relative to onset of NEBD II. From left to right, characteristic still frames from the stages interkinesis (- 42:45), early (06:45) and late (15:45) prometaphase, metaphase (23:15), early (27:45), mid (30:00) and late (33:00) anaphase document that the three univalents present before onset of M II bi-orient normally into a metaphase plate followed by separation of sister centromeres and regular segregation to opposite poles. Scale bar = 2 μm. (TIF) [file pgen.1008928.s005.tif]

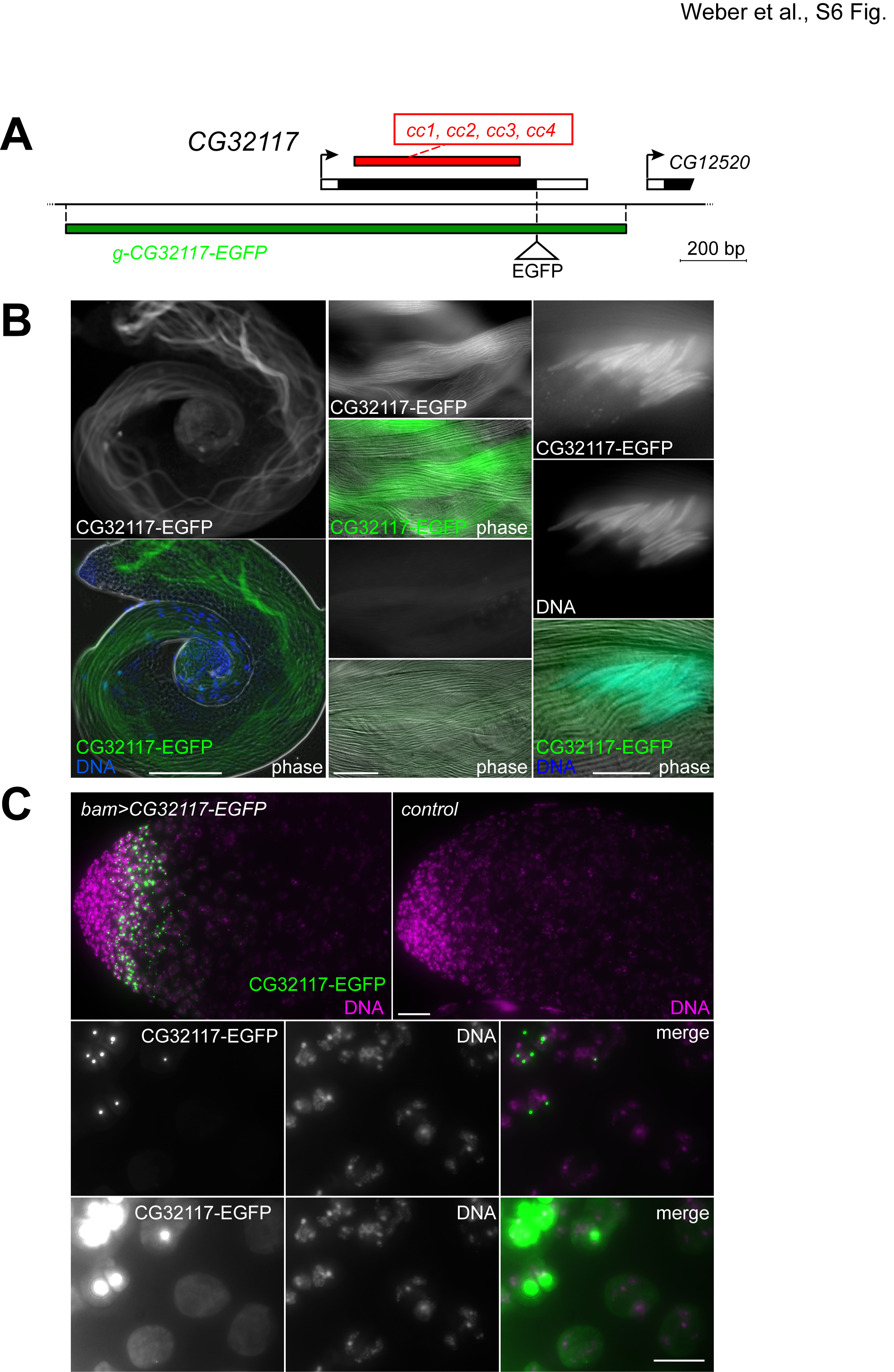

Supplement: S6 Fig — (A) CG32117 transgenes and mutant alleles. The genomic region containing CG32117 is shown schematically. Untranslated regions (white boxes) and coding regions (black boxes) are indicated. The region present in the g-CG32117-EGFP transgene is marked with a green bar. A red bar indicates the regions deleted by the four intragenic out-of-frame deletions induced by CRISPR/cas (cc1, cc2, cc3, and cc4). The precise breakpoints in these four alleles are distinct, although not apparent at the displayed scale. (B) CG32117 expression pattern. Whole mount preparations of testes isolated from either control males (middle column, third and fourth panel from top) and g-CG32117-EGFP males (all other panels) were fixed and stained for DNA. The low magnification view (left column) reveals that g-CG32117-EGFP is expressed in cysts with fully elongated spermatids. CG32117-EGFP signals in the tail region of such spermatids are shown at higher magnification in the middle columns (top two panels), and the comparison with control (bottom two panels) demonstrates that these signals are above the autofluorescent background. CG32117-EGFP signals in the head region of fully elongated spermatids are shown at higher magnification as well (right column). Scale bars = 200 μm (left column), 20 μm (middle column), and 10 μm (right column). (C) Forced premature CG32117-EGFP expression in early spermatocytes. A UASt-CG32117-EGFP transgene was expressed with bam-GAL4-VP16. In the top row, maximum intensity projections of apical testis regions from whole mount preparations stained for DNA are shown. The comparison with control preparations (right) indicates that CG32117-EGFP (left) is clearly detectable but only transiently in early spermatocytes. Higher magnification views from single optical sections with early spermatocytes (middle and bottom rows) illustrate the subcellular localization of CG32117-EGFP. Apart from strong nuclear dots that do not appear to be chromatin-associated (middle row), a wea [file pgen.1008928.s006.tif]

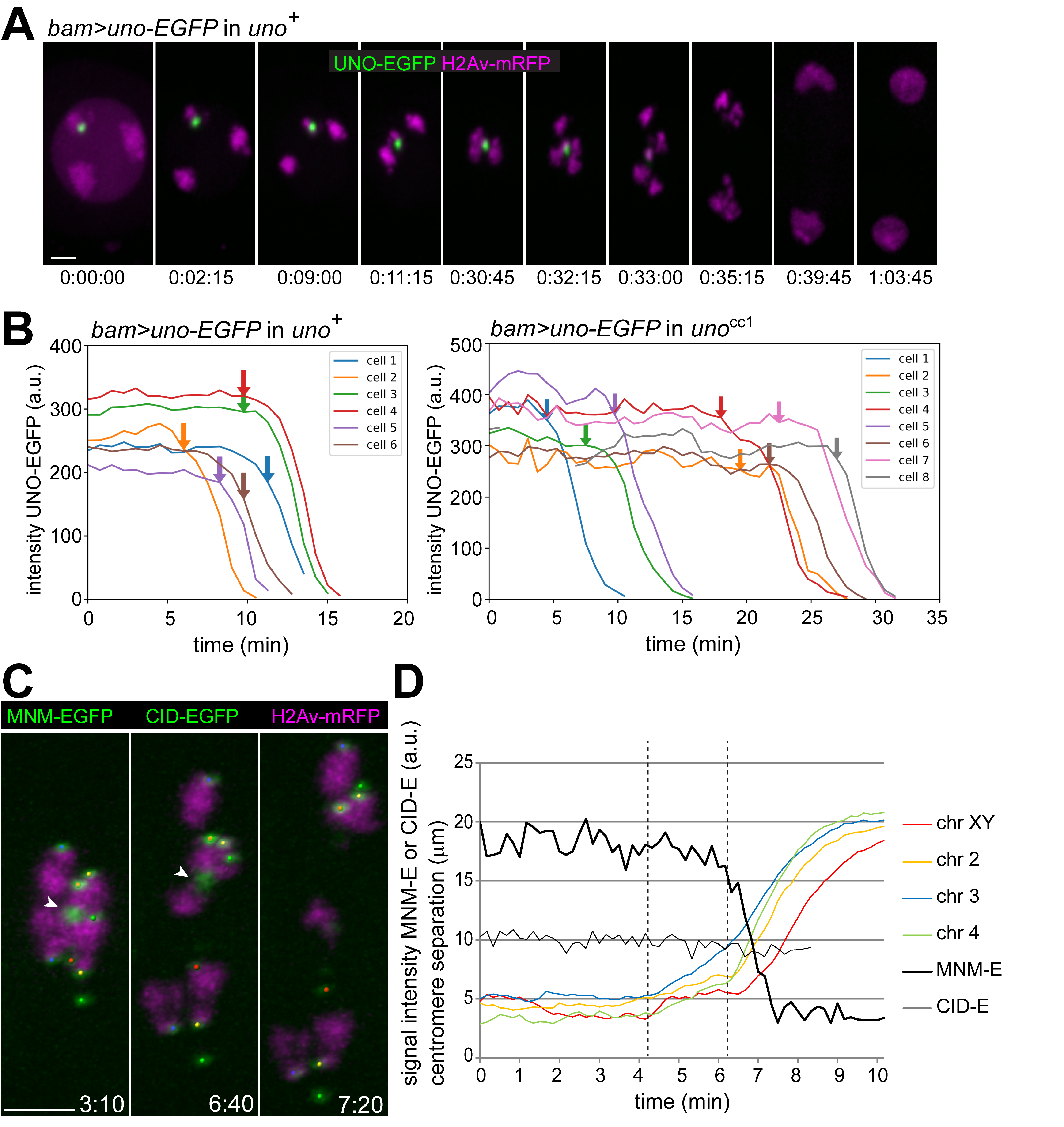

Supplement: S7 Fig — (A,B) Time-lapse imaging with spermatocytes expressing His2Av-mRFP and UNO-EGFP. (A) Still frames illustrate progression through M I in spermatocytes expressing UNO-EGFP in an uno+ background. Time (hours:minutes:seconds) after the last time point before onset of NEBD I is indicated. (B) Quantification of UNO-EGFP signal intensity in the XY-associated dot during progression through M I in spermatocytes expressing UNO-EGFP in an uno+ background (left panel) or instead of endogenous uno+ (right panel). In both panels, several spermatocytes (6 and 8 cells, respectively) were analyzed that were present in the same cyst and therefore divided with high but not perfect synchrony. An arbitrary time point when all spermatocytes were still in metaphase was set to zero. Arrows above the curves indicate the time point when autosomes started to move rapidly and steadily towards opposite poles, i.e., when the final linkages between homologs in the autosomal bivalents were severed presumably. (C,D) Time-lapse imaging with spermatocytes expressing His2Av-mRFP, Cenp-A/Cid-EGFP and MNM-EGFP. The metaphase to anaphase I transition in a representative spermatocyte is documented. An arbitrary time point in late metaphase I was set to zero. (C) Still frames at t = 3:10, 6:40 and 7:20 represent late metaphase before the onset of increased bivalent stretching, an early anaphase stage where autosomes but not the sex chromosomes have started to move apart rapidly, and a later anaphase stage where also the sex chromosomes have started to move apart. Centromeres are marked by colored dots (chr XY red, chr 2 yellow, chr 3 blue, chr 4 green; see [56] for explanation of bivalent identification), the MNM-EGFP dot at the chr XY pairing site with an arrowhead. (D) MNM-EGFP intensity in the XY-associated dot and Cid-EGFP intensity in a selected centromere (to monitor bleaching), as well as the distances between the two centromeres of a bivalent were quantified. The dotted vertical lines mark the time [file pgen.1008928.s007.tif]

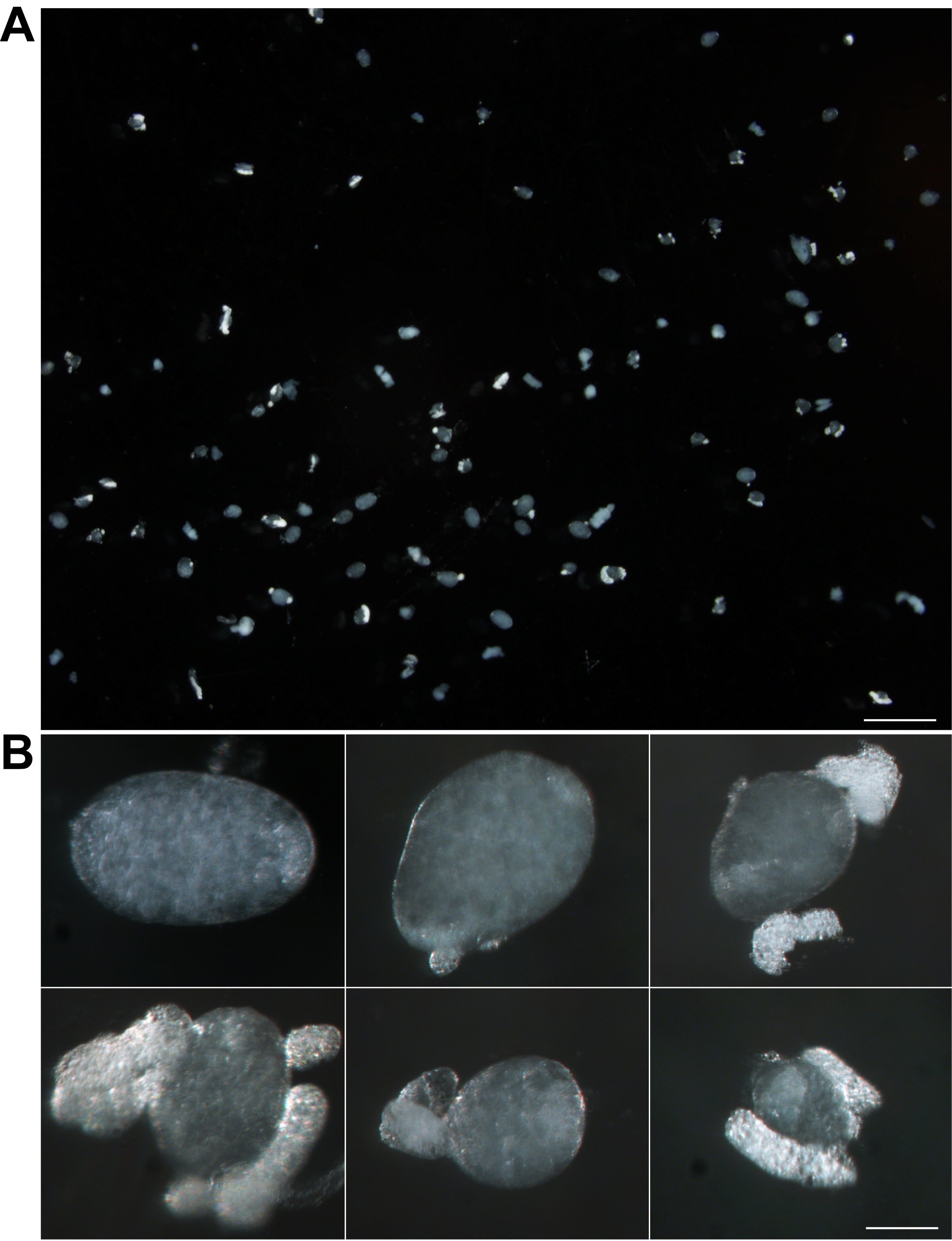

Supplement: S8 Fig — (A,B) Light microscopic images for illustration of the purity of the final testes fraction obtained by mass isolation from late larvae and early pupae. As indicated in Materials and Methods, final testis purity depends critically on the level of the GFP signal in testis and on parameter settings chosen for fluorescent particle sorting. In the optimal case, the fraction of contaminating bits of tissue other than testis is around 3%, as evident from (A). However, testes have residual fat body attachments to variable degrees, as evident from the examples shown in (B), with fat body remnants displaying a characteristic brighter appearance. Scale bars = 1 mm (A) and 100 μm (B). (TIF) [file pgen.1008928.s008.tif]
