# Supplementary material for: Chromosome separation during Drosophila male meiosis I requires separase-mediated cleavage of the homolog conjunction protein UNO
Source: PLoS Genet. 2020 Oct 1;16(10):e1008928. doi: 10.1371/journal.pgen.1008928 (PMC7529252; doi:10.1371/journal.pgen.1008928)
Supplement: S2 Fig — (PDF) [file pgen.1008928.s002.pdf]

Weber et al., S2 Fig.

A

```
Dpse     ------------------------------------MAP---------TLPKSNPSLPAL     15
Dper     ------------------------------------MAP---------TPPKSNPSLPAL     15
Dana     ------------------------------------------------------------     0
Dmel     -------------------------------------MP---------ALTHQ-IL---T     10
Dsim     ------------------------------------------------------------     0
Dsec     ------------------------------------------------------------     0
Dere     ------------------------------------------------------------     0
Dyak     -----------MQPCASFVLTSPFTDRENFVQTFIRLEP---------ALAHQ-II---T     36
Dwil     -------------------------------------------------------MTMQV     5
Dgri     ------------------------------------------------MPTSSAIDTQMM     12
Dvir     MTITSIILFFTYCGCVKFSFIVII-VSELFILIFTQLLKIHSECSARFLQNHQLIYTKKM     59
Dmoj     -----------------------------------------------------------M     1

Dpse     VSKSPRAELSFTIQLLSGRAVVMVECPGYDTELFIPQIVKDRITLQNIMLNRRCCSESS-     74
Dper     VSKSPRAELSFTIQLLSGRAVVMVECPGYDTELFIPQIVKDRITLQNIMLNRRCCSESS-     74
Dana     MASEPRPEVRFDIKLLSAKTVVMVECPGYESELFMPQIVKGRITFQNIIPNRRCCPESI-     59
Dmel     MQRAPPLNLSFKIQLLSGQNVILVECSGYESELFLPQIVGERITMQN1VIQDRRCCAESM-    69
Dsim     MQRASPLNLTFKIQLLSGQNVILVECSGYESELFLPQIVGERITMQNIMQNRRCCAESM-     59
Dsec     MQRAPPLNLTFKIQLLSGQNVILVECSGYESELFLPQIVGERITMQNIMQNRRCCAESM-     59
Dere     MQRAPPKELNFKTQLLSGKNVLLVESPGYESELFMPQIVGGRITLQNVIPNRRCCPESR-     59
Dyak     MQRAPTKELNFKIQLLSGKNVLLVESPDYESELFMPQIVGGRITLQNIIPNRRCCPESR-     95
Dwil     ASKSPRGPLSFNVQLLSGKNVVLVNCQGYESELFMPQIVKGRISFQNIIPNRRCCDQAN-     64
Dgri     ANKTSSGVLQFKIKLLSGNNVVLVDCVGYDSQLFVPQIIKGVITFQNIIPSNRCCNAACG     72
Dvir     PSQTPGAALTFKIKLLSGNNVVLVDCVGFESELFMPQIIKGRITFQNIIPNHRCCDAAAA     119
Dmoj     PSQAPNTALNFKIKLLSGNNVVLVDCVGFESELFMPQIIKGRITFQNIIPNHRCCNAAAA     61
                 : *  :***.. *::*:. .::::**:***:   *::**:: ..***  :

Dpse     -LTTYPTVPP------------VSSGPPVRAP-KRMISSTQPPPVARSTPTSLSV-----     115
Dper     -LTTYPTVPP------------VSSGPPVRAP-KRMISSTQPPPVARSTPTSLSV-----     115
Dana     -MLSEQFVLRGE----VGAGTRLVAGTPLRS----SQAAN-LSPLARSTPSSVTS---LK     106
Dmel     -LLRDPPM-G-D----LGSETRTAKSPPRRF----FAAANPPTPLAKSTPTNTSS---LR     115
Dsim     -MLRDPPM-DMG----MGSGTMTAKSPPRR-----FAAANPPTPLAKSTPTNSSS---LR     105
Dsec     -MLRDPPM-DMG----MGSGTMIAKSPPRR-----FAAANPPTPLAKSTPTNSSS---LR     105
Dere     -MLSDPLM-G-D----MGSRKRTVKSPTRRS----FAAVNLATPKAKSTPINNSS---LR     105
Dyak     -MLSDPVM-A-D----MGSRTRIAKSPPRRS----FAAANPPTPLAKSTPMNTSS---LR     141
Dwil     -IITGPLPLNGAAC----IGT----GGKPTLAGTPAIQRPTSISLARSTAMRSRP-KRSK     114
Dgri     QGQKGQFILPATKFGNVRIVPPL------GYNSGVDVS-NKSAPLARSTPSRGSS-----     120
Dvir     NVQTGQLILPASKLGDVRVVAGA--GTPQRFNLEQHIA-NISGPLARSTPSSGT-SVRAV     175
Dmoj     NVQTGQLILPASKLGDVKIVAGIAAGAAKRLNYQPHVA-SKTSPLARSTPSSSSSSERAI     120
                                                      *:**

Dpse     RRIRAANKTVELTPEPARSPAIPTSLSMRRIRAANKTVELTPEPARCQAIPFESEIQ---     172
Dper     RRIRAANKTVELTPEPARSPAIPTSLSMRRIRAANKTVELTPEPARCQAIPFESEIQ---     172
Dana     SRASREKN----------------------------L-HATPELVRCRPMKHSS------     131
Dmel     ARAGKENA----------------------------VPQLTPEGLRCRPFHLPA------     141
Dsim     ARAGKENA----------------------------VPQLTPEGLRCRPFHLSA------     131
Dsec     ARAGKENA----------------------------VPQLTPEGLRCRPFHLSA------     131
Dere     TRAGKENA----------------------------VPQLTPERLRCRPFHLPS------     131
Dyak     SRAGKENA----------------------------VPQLTPEGLRCRPFRLPS------     167
Dwil     ETISPPPH----------------------------GAIMTPDQLRGKPAEVEF-----S     141
Dgri     VRAEKENV----------------------------SMVLTPEQTRCRPTQMMT----SP     148
Dvir     IRADKENF----------------------------SLLLTPEQARCKPVQKS----ALP     203
Dmoj     SRADKENL----------------------------SMQMTPEQVRCRVPAGTGQMAALP     152
                                                 **:  * :

Dpse     --------DVSLHVNQSYSNSSL---------------ESPPPLLPPE------------     197
Dper     --------DVSLHVNQSYSNSSL---------------ESPPPLLPPE------------     197
Dana     -LGEVEIDLGEGYM-PPDLEVTTGSLPS--------------------------PGARLS     163
Dmel     -PVEMDVDQLCCQEQTPERRASSKSISSANTNNSFFENEVSQPVISSK---NVYANESAR     197
Dsim     -PGEMDVDELCCEEQTPERRSSSKSIGSANTNNCFFENEISQPEISSKDVTNVYVNDLGR     190
Dsec     -PGEMYVDELCCEEQTPERRSSSKSISSANTNNCFFENEISQPEISSKNVTNVYANDLGR     190
Dere     -DGEMDIDELSCQEQAPGSLSSSKSMMSACTNNSLSEIEVLSPVISSKNGSNVHANELGR     190
Dyak     -QGEIHMDELSCQERAPGSVASSKSMMSAYTNNSLSQTEMTSPVILSKNVTYVHANESGR     226
Dwil     IPEDLNPEEL---------FSSFYAE-----LPNQLRL------------------SDGR     169
Dgri     TSATINS-DT-----QPFLESSFHTC-----NLPISELPDVSPLRPVA------NTPDSS     191
Dvir     IP--VSIDSI-----LPFLESSSAA------------SPVANPSLP----------VANV     234
Dmoj     TPPPICLDAL---------ETSSTST-----VPNLALESYTNRTVT----------QDSF     188
                              :
```

```
Dpse      ----VLSITDAMM------SVSSTPNFNLNASPPLTSSMRVKPPERTYARRKTNNIG---      244
Dper      ----VLSITDAMM------SVSSTPNFNLNASPPLTSSMRVKPPVRTYARRKTNNIG---      244
Dana      STYHSPSLPESKLSPPAGPSYNLVAAQLKE---SLSNITQKNPVLRTYSRKKAESSG---      217
Dmel      NNITPSSIYETGISPPVLPRENLTEQNHQAMELAINKTKPKLTAVRTYTRKRVETAS---      254
Dsim      NNITPLSISETGISPPVLPKENLTEQNHQPMELAINKTRPKLTAIRTYTRKRADTAS---      247
Dsec      NNITPLSISETGISPPVLPKENLTEQNHQAMELAINKTKPKLTAVRTYTRKRADTAS---      247
Dere      NNFTPLSISETRIPPPVMPKENVIEQNLQAMEVVHSKSKPKVSALRTYSRKKPGTAS---      247
Dyak      KIFTPLSISETRISPVVLPNETYTEQNPQAIEVVHNKSKPKVAAVKTYSRKKAGTAT---      283
Dwil      N---------------ELNPGSSTHTPAQSLESTISSVIRKTPPVRTYSRRKTNATS---      211
Dgri      LR-------------RRLASTASSPDSATTSEPALYNFMHKAPPLRTYSRRKGDTTT---      235
Dvir      SA-------------PRPTSTSSSALSAKTGDTALCNFMHKAPPVRTYARRKPNTTST--      279
Dmoj      NV-------------IEPPSTSSLSSSAKSPDFAQCNFMHKAPPMRTYSRRKPNTTDTNN      235
                                              .        :**:*::

Dpse      ------------KAKTPPKWSPVQNKKQQ--LLSATRGPNPSMHIEVRRRNFLEDRHKTI      290
Dper      ------------KAKTPPKWSPVQNKKQQ--LLSATRGPNPSMHIEVRRRNFLEDRHKTI      290
Dana      ------------VAK-ATVWSPINKKKK-EKI-SLTKAPSPTVKVEVRKRKLIVDTKKSI      262
Dmel      ------------KAKATPVWSPLQKKKKSSWV-SSTKVPSPTMKVEVRNRKLIVDTKKTL      301
Dsim      ------------KAKPTPVWSPLQKKKKNSSV-SSTKVPSPTMKVEVRKRKLIVDTKKTL      294
Dsec      ------------KAKPTPVWSPLQKKKKNSSV-SSTKVPSPTMKVEVRKRKLIVDTKKTL      294
Dere      ------------KAKPTPVWSPLQKKKKNSSV-SSTKVPSPTMKMEVRYRKLIVDTKKTL      294
Dyak      ------------KAKPTPVWSPLQKRKKNSSV-SSTKVPSPTMKVEVRNRKLLVDTKKTL      330
Dwil      ------------KSKSSLSCSPLQKKKKISSKASTTKGLQATKTLAIRNRRLIVDNKKSL      259
Dgri      ------------QSTASISWSPLSKKKRLAKV-SSTKPPSPRLKMEVRHRKLIVDQKKTL      282
Dvir      ------------QPKGPISWSPLQKKKKLAKV-SSTKPPSPSMKLEVRHRKLIVDQKKSL      326
Dmoj      SSGGGSSSASVSHPKSPLAWR----KKKLTKV-SSTKPPSPSMKLELRRRKLIVDQKKSL      290
                        .          :*:     * *:  .    : :* *.:: * :*::

Dpse      EPYDLQNTVSSPVVQKEIIQRQVTAKTKQQFDALKITAFDLLTTSAQGKIAEELVQQFQE      350
Dper      EPYDLQNTISSPVVQKEIIQRQVTAKTKQQFDALKITAFDLLTTSAQGKIAEELVQQFQE      350
Dana      AAHDPNKIAPIPITKKKIIRKQVTGKTRKQFEALKATAYDLLTNPSLSHMSSDLCKQFQL      322
Dmel      AVHDPRKLSSGSITMKKIIRKQVTGKTRKQFEALKVTAFDLLTNLCVANISDDLTKQFQK      361
Dsim      AVPDPRKLSSGSITMKKITMKQVTGKTRKQFEALKVTAFDLLTNLCVSNISEDLNKQFHK      354
Dsec      AVHDPRKLSSGSITMKKIIMKQVTGKTRKQFEALKVTAFDLLTNLCVSNISEDLNKQFHK      354
Dere      AANDPSKLSPGPITMKKIIRKQVTGKTRKQFEALKVTAFDLLTNLCVGNISEDLVKQFQK      354
Dyak      AAHDPSKLSAGPITMKKIIRKQVTGKTRKQFEALKVTAFDLLTNLCVGNISEDLAKQFQR      390
Dwil      GSLSVSK-LSSSITKKKTVHNQVTAKTRKQFESLKTTAFDLLTTPTLENISKTLNQQFQM      318
Dgri      SQPDAPT-LSIPIIKTKIQGKLVSAKTRRQFEALKLTAFDLLTSPGISRLSSELYQQFKD      341
Dvir      AATDTHKITAVPITKTKIRRKQVTGKTRQQFEALKGTAFDMLTSPGHSHVSIELSKQFKD      386
Dmoj      PKHTYTKITATPITTTKIRRKQVTGKTRQQFDALKGTAFDMLTSMGHSHVSVKLTKQFKE      350
                .     :  .:   . *:.**::**::** **:*:**.    .::  * :**:

Dpse      ACIDRRSSTVPNYIQLSGSSQLQLDHEVKTLLARQKRLDYVHKKPLKWMKRM----          402
Dper      ACIDRRSSTVPNYIQLSGSPQLQLDHEVKNLLARQKRLDYVHKKPLKWMKRM----          402
Dana      ACMDKCATTLPNYAEVAVTPPLELDRQVKSLMAKQKRMDRKEQIPKSVTSIMGKRI          378
Dmel      ACANRVCTTLPNYAEIAVVPPLEMDREVNALMARQKRMERETHRRWSAVPPEGNPV          417
Dsim      ACANRVCTTLPNYAEIAVVPPLEMDREVNALMARQKRMERETQRRRSAVPPECNPA          410
Dsec      ACANRVCTTLPNYAEIAVVPPLEMDREVIALMARQKRMERETQRRWSAVPPECNPA          410
Dere      ACANRVCTTLPNYAEIAVVPPLEMDREVTAIMARQKRMERGSQRPRSAIPLDSNLA          410
Dyak      ACANRVCTTLPNYAEIAVVPPLEMDREVTAMIARQKRMERDCRRPRTAMPLERNLA          446
Dwil      ACVDKIATTLPNYAQLAPTKPLEIDREVKTMIAKQKRLEKTNSKPKWI--------          366
Dgri      ACVDRYCETLPSYVELSSVAPLQQDRQVKTLLAKKKRLERQLTKSEQARLIKGRRV          397
Dvir      ACVEKYSETLPSYAELSGTAPLEHDRQVKSLIAKEKRLEKQMSKSEQARLLKRKRI          442
Dmoj      ACVEKYSEALPSYAELSGTRPLEHDRQVRSLIAKAKRLDKQMSKSEKVQLKRKRM-          405
          ** :: . ::*.* :::    *: *::*  ::*: **::
```

B

```
Dmel 323  QVTGKTRKQFEALKVTAFDLLTNLCVANI-SDDLTKQFQKACANRVCTTLPNYAEIAVVP      381
Ccap      -VTGKTRKQFEALKVTAFDLLTNLCVANI-SDDLTKQFQKACANRVCTTLPNYAEIAVVP
Lcup      ----KPRPLRETMALSAFDLLTNSNHQRCMSAKLNEQFRRGCVNKASSTYSKYKVLSTVP
Mdom      ---------------SAFDLLTNSNRSQCMSNKLKELFRERCVNKASSTYSKYKVLCNIP
Scal      ---------------SAFDLLTNSNRVACLSSKLTEQFRKGCINKASSTNSKYKILANVP
Rzep      -----DRRQYDMSKKTAFDMLTAPSRSH-VATKLAQQFKKACVNKASSTCPKYKILCKIP
Zcuc      -TTAKDRKKYEVNKKTAFDMLTAPSRTPMMSSKLTQQFKKACVNKASSTCPKYKILCKIP
Bole      -TAAKDRKKYDINKKTAFDMLTAPSR-PVMSSKLSQQFKKACVNKASSTCPKYKILCKIP
Blat      -TTAKDRKKYEMNRKTAFDMLTAPSI-PVMSSKLTQQFKKACVNKASSTCPKYKILCKIP
Bdor      -TAAKDRKKYEINRKTAFDMLTEPSR-PVMSSKLTQQFKKACVNKASSTCPKYKILCKIP
                         :***:**        : .* : *:. * *:..:*  :*  :. :*

Dmel      PLEMDREVNALMARQKRMERETHRR      406
Ccap      PLERE--------------------
Lcup      SLQQDEQVLEL--------------
Mdom      PLQEDEQVLEL--------------
Scal      P------------------------
Rzep      PLEQPERQPKGKYSVNKYL------
Zcuc      PLEKVEKNRNGERRDGNIDR-----
Bole      PLEQVEESRNREHRDVNIDRK----
Blat      PLEQVEESRN---------------
Bdor      PLEEVEESRN---------------
```

S2 Figure. Multiple amino acid sequence alignment of putative UNO homologs.

(A) The predicted *D. melanogaster* UNO amino acid sequence was used to search for homologous proteins in sequenced genomes of *Drosophila* species (using blastp at http://flybase.org/blast/). Clustal Omega was used for the generation of a multiple sequence alignment of the amino acids sequence of the identified UNO homologs (https://www.ebi.ac.uk/Tools/msa/clustalo/). Amino acid sequences are given in one letter code and a color code for chemical character. Positions with an identical amino acid in all the aligned sequences are marked with an asterisk below the aligned sequences, and with a semicolon at positions with similar amino acids. The more strongly conserved N- and C-terminal regions are indicated by highlighting in yellow and turquoise, respectively. A conserved region including a match to the separase cleavage site consensus (Sullivan et al. (Uhlmann) 2004. JBC. Studies on substrate recognition by the budding yeast separase; Lin Z, Luo X, Yu H. 2016. Nature. Structural basis of cohesin cleavage by separase) is highlighted in grey. Dper: *Drosophila persimilis*, Dana: *Drosophila annanassae*, Dmel: *Drosophila melanogaster*, Dsim: *Drosophila simulans*, Dsec: *Drosophila sechellia*, Dere: *Drosophila erecta*, Dyak: *Drosophila yakuba*, Dwil: *Drosophila willistoni*, Dgri: *Drosophila grimshawi*, Dvir: *Drosophila virilis*, Dmoj: *Drosophila mojavensis*

(B) A region of *D. melanogaster* UNO (amino acids 323-406) which covers the most conserved part close to the C-terminus was used to search for homologous proteins within the non-redundant protein sequences database (at https://blast.ncbi.nlm.nih.gov/Blast.cgi using blastp and PSI-BLAST). Clustal Omega was used for the generation of a multiple sequence alignment of the identified amino

acids sequences which suggest that UNO homologs might be present in species of the higher dipteran families of Tephritidae, Calliphoridae and Muscidae. Dmel: *Drosophila melanogaster*, Ccap: *Ceratitis capitata (*Tephritidae*)*, Lcup: *Lucilia cuprina (*Calliphoridae*)*, Mdom: *Musca domestica (*Muscidae*)*, Scal: *Stomoxys calcitrans*, Rzep: *Rhagoletis zephyria (*Tephritidae*)*, Zcuc: *Zeugodacus cucurbitae (*Tephritidae*)*, Bole: *Bactrocera oleae (*Tephritidae*)*, Blat *Bactrocera latifrons (*Tephritidae*)*, Bdor: *Bactrocera dorsalis (*Tephritidae*)*
