# Supplementary material for: Chromosome separation during Drosophila male meiosis I requires separase-mediated cleavage of the homolog conjunction protein UNO
Source: PLoS Genet. 2020 Oct 1;16(10):e1008928. doi: 10.1371/journal.pgen.1008928 (PMC7529252; doi:10.1371/journal.pgen.1008928)
Supplement: S3 Fig — (PDF) [file pgen.1008928.s003.pdf]

A

```
CG32117:
MTSFQIRLISDQNLVLVDALHHESEIFVPQIYNNDIVLWNIIRDRRSNPKVEFCEEPVQGQGNGQICQRPQIQPRITTATAGL
APWAASIDGSPFRRVAEEGSRCPLFSLKLISNGSVVLVECNVHESEIFLPQICGQYIAMKRITGHELLLPSKPKISAIQPAKT
NCLRIQPTRFFSKTTNLDNQAKKRRKRKSQEQTNHSGAGDQRTLKSVKR
```

B

```
UNO_Nterm cons  20 SFKIQLLSGQNVILVECSGYESELFLPQIVGERITMQNVIQDRRCCAESMLLRDPPMGDLG 80
                   SF+I+L+S QN++LV+   +ESE+F+PQI    I + N+I+DRR   +     +P  G
CG32117_rep1     3 SFQIRLISDQNLVLVDALHHESEIFVPQIYNNDIVLWNIIRDRRSNPKVEFCEEPVQG  60
```

C

```
UNO_Nterm cons  20 SFKIQLLSGQNVILVECSGYESELFLPQIVGERITMQNVIQDRRCCAESMLLRDPPMGDLG 80
                    F ++L+S  +V+LVEC+ +ESE+FLPQI G+ I M+ +        E +L   P +  +
CG32117_rep2   107 LFSLKLISNGSVVLVECNVHESEIFLPQICGQYIAMKRITGH-----ELLLPSKPKISAIQ162

UNO_Nterm cons  81 -SETRTAKSPPRRFFA  95
                    ++T   +  P RFF+
CG32117_rep2   163 PAKTNCLRIQPTRFFS  178
```

D

```
Dana      ---------MTNFRIQLLSDNNVVLVQAIGHETEIFLPQLYNERFALRNIIRNRRPNARY      51
Dsec      ---------MTSFRIRLISDQNLVLVDAPHHESEVFVPQIYNNDIVLWNIVRDRRSNPKV      51
Dmel      ---------MTSFQIRLISDQNLVLVDALHHESEIFVPQIYNNDIVLWNIIRDRRSNPKV      51
Dsim      ---------MTSFRIRLISDQNLVLVDAPHHESEVFVPQIFNNDIVLWNIVRDRRPNPKV      51
Dere      ---------MTSFHIRLISDKKVVLVDASHHESEIFVPQMHQTGIVLWNIIRNRRPNPKL      51
Dyak      ---------MTSFQIRLISDKNVVLVDALHHESEIFVPQLHQNDIVLWNIIRNRRPNPKL      51
Dwil      MPKRMMSTNPLPFRIQLLSGRSVVLVEEPSHESEIFLPQLLHGRIVLENVVHSRQRRLRE      60
Dgri      MCKRCTTVSPHKFDIRLLAEKSVILVETPGHESEIFLPELQHGRVTLSNIIRSRQPSVRP      60
Dvir      MCEHSATTSHIRFNIQLLADKSVVLVEARGYESEIFVPVLQHGRLCLTNVICSRQPRVRA      60
Dmoj      MCNSTESTNKSRFQIQLLADRSVVLVNARGYESEIFLPELLKGRVCLRNVICSRQPKSPP      60
                     .* *:*:: ..::**:   :*:*:*:* : :  . * *:: .*:

Dana      DAGEDVPNQGQLPSSHPIASH--------PNQRPRLTTATAGLAPMGESPPPGGGRGSST      103
Dsec      EFCEEPV-QGQD-NGQL-CQR--------PQIQTRITTATAGLAPWAVSIEGSP------      94
Dmel      EFCEEPV-QGQG-NGQI-CQR--------PQIQPRITTATAGLAPWAASIDGSP------      94
Dsim      EFCEEPV-QGQD-NGQI-GQR--------PQIQTRITTATAGLAPWAVSIDGSP------      94
Dere      EFCDEST-HGQD-NGQV-VQR--------PQMQARITTATAGLAPWAASIDGGA------      94
Dyak      QFCEESI-HGQD-NGPVVLQR--------PRIQTRITTATAGLAPWAASTDGGA------      95
Dwil      SETN--L-AGR--RLRQNCRSQRLMGGGGSGGQQKVTTATVGLAPLAIMDNGLMLQSQLQ      115
Dgri      SGVC--L-HST--LSSHPGQR---------LANRKLNTTMSDLAPKSS-----G------      95
Dvir      SELS--F-HSP--TSLHRCQR---------QANRKLTTATAGLAPKKLPSVSIA------      100
Dmoj      SEMS--S-QSS--SSDLRCPR---------IRNLKLTTATAGLAPNPR------------      94
          .        .                      . ::.*:  .***

Dana      YSGASGGGGGGGEGSDHARPLQFTLKLISNEKVVLVESSGHESEIFLPYLSGQFIAMKRV      163
Dsec      ---------FRRIAEEGSRCPLFSLKLISNGSVVLVECNVHESEIFLPQICGQYIAMKRI      145
Dmel      ---------FRRVAEEGSRCPLFSLKLISNGSVVLVECNVHESEIFLPQICGQYIAMKRI      145
Dsim      ---------FRRIAEEGSRCPLFSLKLISNGSVVLVECNVHESEIFLPQICGQYIAMKRI      145
Dere      ---------FRRVADEGVRCPQFSLKLISNGSVVLVECNVHESEIFLPQVCGQYITMKRI      145
Dyak      ---------FKRVADEGSRCPQFSLKLISNGSVVLVECNVHESEIFLPQICGQYMAMKRI      146
Dwil      FQ--------LQQKQQQLQHPCITLKLILNGKVVLVECNGYESEIFLPHICSHCIAMKRV      167
Dgri      -------------RDRNVSKLRFELKLISNGNVVLAECNGYESEIFLPHIFSHCVTMKRV      142
Dvir      -------------RAGDVSKLRFELKLISNGNVVLVECNGYESEIFLPQISSHCVAMKRV      147
Dmoj      -------------RRDNTSRLRFELKLISNGKVVLVECNGYESEIFLPHIRSRCVAMKRI      141
                               .: **** * .***.*.. :******* : .: ::***:
```

```
Dana      TANELIQCSRLKSRPQLTPNG--ALNQPQALPPNYPHQDHLSKRDQLFFNPSQFFFHNNS    221
Dsec      TGRELLLPSKPKISA-VQP----------------------AKSNCLRIQPTRFCSKTTN    182
Dmel      TGHELLLPSKPKISA-IQP----------------------AKTNCLRIQPTRFFSKTTN    182
Dsim      TGRELLLPSKPKISA-IQP----------------------AKSNCLRIQPTRFCSKTTN    182
Dere      TAHELVLPSKPKMPA-IQP----------------------AKTDCLRVQATRFFSKTTN    182
Dyak      TGHELVLPSQPMMPA-IQP----------------------AKTNCLRLQSTRFSSKTTN    183
Dwil      TANQLVQVQNSHKRPSNKAPSSYASTAAEC-SP-Y--NPSLNQANRMLIHPMQMLDRHAE    223
Dgri      SAGQLAQYAECRKSKRNQSSSESANNAASTAAAYL--VAHADNQTKLLVKPIHLIMNTSD    200
Dvir      SAGELAQCVSRKPKKERTSTKNTAAF----MAATL--ATQAVGLSKLLVNPLHLFYKEEK    201
Dmoj      SAKELAQCASAKSSRSQCTQK-SPNN----SAASL--ATQAVGLSCLWPTSPKSGNVIDK    194
          :. :*                                         :     :      .

Dana      TDTNTHES--------EKQVKTRRKRR-------------YQPKYAGAGDQQSAGKR---    257
Dsec      LD---------------IQAKKRRSAKVK-----------SKPITLELGINER-------    209
Dmel      LD---------------NQAKKRRKRKSQ-----------EQTNHSGAGDQRTLKSVKR-    215
Dsim      LD---------------IQAKKRRKRKSQ-----------EQTNHSRAGDQRTLKSAKR-    215
Dere      LD---------------IQARKRRKRKSQ-----------EQTNHSGAGDQPKSKSLKR-    215
Dyak      LD---------------SQAKKRRKRKSQ-----------EPAHHSGAGDQPVSKSVKR-    216
Dwil      NS--IAF----GKEQLLKLSKKERNRRRR-LNYNQNQNHMKQ-----SGAAAAVALESPI    271
Dgri      SQNEQKYQE----KQIVNNVNK--GSKVKVPSVQSKKKHRKQILHKAAGDA---------    245
Dvir      EQKEQKEHKGSQEGKSQKAHKEPMEQKEKLSPVKQKKRHVKH-NSKEAGDA---------    251
Dmoj      QKSGQR----IQKGQQVKKMKNPLKDKSS---TRQKKKRCKQ-YSKETRDVSN-------    239
           .                  .     :
```

S3 Figure. *CG32117* codes for an UNO related protein

(A) The complete predicted *D. melanogaster CG32117* amino acid sequence is shown with color highlighting indicating two highly similar regions (repeat 1 turquoise, repeat 2 magenta).

(B,C) The *CG32117* repeat regions are highly similar to the conserved region close to the UNO N-terminus (yellow highlighting, as in S1 Fig.). Alignments of this UNO region with *CG32117* repeat region 1 (turquoise highlighting, as in A) and *CG32117* repeat region 2 (magenta highlighting as in A) are shown in (B) and (C), respectively.

(D) The predicted *D. melanogaster CG32117* amino acid sequence was used to search for homologous proteins in sequenced genomes of *Drosophila* species (using blastp at http://flybase.org/blast/). Clustal Omega was used for the generation of a multiple sequence alignment of *CG32117* homologs (https://www.ebi.ac.uk/Tools/msa/clustalo/). Amino acids in one letter code and a color code for chemical character. Positions with an identical amino acid in all the aligned sequences are marked with an asterisk below the aligned sequences, and with a semicolon at positions with similar amino acids. Repeat regions 1 and 2 highlighted as in (A-C). While searches for *CG32117* homologs within the non-redundant protein sequences database (at https://blast.ncbi.nlm.nih.gov/Blast.cgi using blastp and PSI-BLAST) resulted in hits from trephritid and muscid dipterans, these were found to represent *uno* homologs. Therefore, compared to *uno*, the taxonomic distribution of *CG32117* like genes appears to be more restricted (to *Drosophila* species). Dana: *Drosophila annanassae*, Dsec: *Drosophila sechellia*, Dmel: *Drosophila melanogaster*, Dsim: *Drosophila simulans*, Dere: *Drosophila erecta*, Dyak: *Drosophila yakuba*, Dwil: *Drosophila willistoni*, Dgri: *Drosophila grimshawi*, Dvir: *Drosophila virilis*, Dmoj: *Drosophila mojavensis*.
