# Supplementary material for: Chromosome separation during Drosophila male meiosis I requires separase-mediated cleavage of the homolog conjunction protein UNO
Source: PLoS Genet. 2020 Oct 1;16(10):e1008928. doi: 10.1371/journal.pgen.1008928 (PMC7529252; doi:10.1371/journal.pgen.1008928)
Supplement: Attachment — Submitted filename: Response to Reviewers.pdf [file pgen.1008928.s024.pdf]

Response to reviewers

We would like to thank all of the reviewers for their insightful and constructive comments.

We have submitted a manuscript version where all of the changes that were made during revision are indicated in the edit mode. However, this version with tracked changes is based on a file before final formatting of references and deletion of the field codes. As a result, line numbers in this version refer to different text compared to those in the final revised version that we have submitted. Line numbers given below refer to the final revised version.

<u>Reviewer #1</u>

We have numbered the points of minor criticism to which we have responded as follows.

1. The authors' method for purifying testes expressing MNM-GFP or SNM-GFP seems to have worked at least fairly well since they found UNO. But the same argument does not hold for the eGFP control, making it more difficult to assess what value the control provided. Moreover, beyond UNO, the value of the list of interactors in Table S1 to the community at large depends on how pure the testis preparations were, which is not mentioned. In addition to final purity, it would be helpful to investigators who wish to try this method to provide information about how well the major steps in the testis purification protocol worked. It would also be helpful to detail, if possible, whether and to what degree the proteins recovered exhibit a bias toward testis-specificity or enrichment and a bias against specific expression in other organs.

-> We have added further information to the revised description of our testis mass isolation procedure in Materials and Methods (lines 1013-1016, 1032-1048), as well as an additional supplemental figure (S8 Fig).

We agree that it would be useful to have additional information providing further hints as to which of the many proteins identified in the MNM-EGFP and SNM-EGFP pulldown experiments might be specific MNM and/or SNM interactors rather than non-specifically associated contaminants. However, further bioinformatic analyses concerning testis enrichment is unlikely to provide helpful evidence as our pulldown experiments were performed with extracts prepared after testis purification. Thus, information whether a particular identified proteins is testis-specific or testis-enriched cannot resolve whether its association with the baits is specific or non-specific. Similarly, proteins, which are not testis-specific (or not testis-biased), can be specific and non-specific interactors in principle.

2. Line 1197 & Fig 2B Contrary to the statement, I cannot see 8 univalents in PMI in the RNAi cell although I can see them in the uno- mutant.

-> Indeed the number of chromosomes that are clearly distinguishable in squash preparations in *uno* mutant and *uno* depleted spermatocytes is not always eight in prometaphase I. According to time lapse imaging, some bivalents appear to have still a rather normal appearance during NEBD I before they are separated prematurely into univalents by spindle forces during prometaphase I. Moreover, distinct chromosomes might cluster on top of each other in squash preparations. Therefore, we have corrected the legend of Fig 2B. It now states that "an increased number of chromosomes is detected ..." (line 291-292).

3. Fig. 2D & E It is very hard to distinguish the red+green dots from the green and red ones in the mutant. It would help greatly to present side-by-side images of the separate fluorescence tracks and a merge image without the DNA. This figure is important because it is the only actual evidence for the claim that the X and Y chromosomes segregate randomly. The quantification is also incomplete in that it does not show the actual numbers of each class of nuclei. The authors' hypothesis predicts not only an equal ratio of regular and nondisjunctional spermatids but also an equal ratio of XY and nullo-XY spermatids. An excess of nullos could indicate that there is some meiosis II nondisjunction as well.

-> To ascertain correct identification of the different types of spermatid nuclei in Fig 2D, we have added yellow arrowheads (indicating abnormal nuclei without any FISH signals) and yellow arrows (indicating abnormal nuclei with both a green and a red FISH signal). We are confident that the quality of the merged images in the final figure will allow an independent visual confirmation of these assignments. We would like to point out that beyond Fig. 2D,E, our manuscript includes additional support for the conclusion that sex chromosome segregation is random during M I in *uno* mutants. Time lapse imaging (Fig. 2F,G) clearly revealed that all bivalents including the sex chromosome bivalents separate prematurely, resulting in random segregation of univalents during M I in *uno* mutants.

In *uno* mutants, the average ratio between spermatid nuclei without FISH signals and spermatid nuclei with both a red and a green FISH signal is 0.95. We have amended S4 Table, in which the complete quantification was reported already in the original submitted version. For convenience, the revised version now contains an additional column in which the resulting ratios are calculated. The observed average ratio of 0.95 is close to the ratio = 1 expected in case of random segregation of X and Y during M I followed by regular sister segregation during M II and considerably lower than the expected ratio = 2.77 in case of random segregation of X and Y during M I and random sister segregation during M II. Our results XY FISH results agree with our time-lapse imaging and support our conclusion (random segregation of univalents during M I and regular segregation during M II). To prevent readers from overlooking this supporting additional evidence in favor of regular M II segregation, we have extended the text describing M II segregation in *uno* mutants (line 398-411).

4. Relative to the issue of how the X and Y segregate, the most direct approach would be to score anaphase I and telophase I nuclei for distribution of the red and green FISH spots. This isn't any harder than scoring spermatids and is more direct.

-> We agree that an analysis of anaphase/telophase I after XY FISH gives direct information concerning sex chromosome segregation, but for us this is definitely harder than scoring early spermatids. Cysts with early spermatids are substantially more frequent than anaphase/telophase I figures and the early spermatid cysts can be analyzed rapidly with our Image J pipeline.

Random segregation of sex chromosomes during M I in *uno* mutants is actually also indicated by our M I time lapse analyses, as they clearly revealed premature separation of all bivalents followed by random segregation of univalents. Overall we consider our evidence to be strong and conclusive even without scoring anaphase/telophase I after XY FISH.

5. Fig. 2F is very clear and it is easy to see how the images could be used to assess the overall segregation pattern. But actual numbers of cells scored should be provided for Fig. 2G (and 2C and 2E as well).

-> The actual numbers were all given in the original S4 Table. In the revised version, these numbers are now also provided in the figure legend (lines 308, 325).

6. In Figs 2C and 2E the meaning of the error bars needs clarification. I think each cyst is treated as a separate experiment and the error bars represent the differences between the averages for each cyst, but I'm not sure. Also did all the cysts come from one testis or were different males sampled?

-> The interpretation suggested for the meaning of the error bars is correct. We have further clarified our explanations for the error bars in the figure legends (lines 299-301, 308-309). We cannot specify the number of males that were analyzed precisely because for each slide of testis squash preparation, testes dissected from 5-6 males were pooled. However, we routinely analyze at least two slides per genotype, making sure that that conclusions are never based on analysis of only a single male. We have added this information into the Materials and Methods section on microscopy (lines 1118-1119, 1127-1128).

7. Fig. 3A - At the low magnification shown and in the absence of stage markers, it is hard to tell whether UNO signals are present in spermatogonia. Can the authors provide more definitive evidence to support their claim that UNO is expressed only in spermatocytes?

-> As we have not used stage markers, we cannot completely exclude that UNO accumulation might start already in late spermatogonial cells. Accordingly, we have qualified our statement accordingly. (line 436-437).

8. Fig. 4B - It is not clear exactly what the arrows in the SNM nucleus are pointing at: UNO or SNM-eGFP or both? Presenting individual images of the separate tracks would clarify.

-> As suggested, we have added additional insets of single channel images into Fig 4B.

9. Fig. 4C - there appears to be some weak staining of autosomes in the tef- overexposed image. The quantification to the right focuses on spot numbers but much of the staining is diffuse. Is there a way to express the overall difference in staining of autosomes between tef- and tef+?

-> Unfortunately, even in *tef*+ the dot-like autosomal UNO-EGFP signals are weak and not reliably detectable above the diffuse background in each S5 spermatocyte (and if detectable not necessarily in both large autosomal territories). Similarly, the slight and diffuse enrichment of the very weak UNO-EGFP signals on the large autosomal territories in *tef*- are only apparent in some S5 spermatocytes and not consistently in both large autosomal territories. We have added additional single UNO-EGFP channel images to Fig 3C and Fig 4C for optimal comparison between *tef*+ and *tef*-.

10. Fig. 7 & movie S9 – the ability of the autosomal bivalents in the uno+ background to segregate despite being, presumably, held together by uno[nc] is surprising. The authors point out this interesting behavior but do not offer an explanation. I'm curious whether it is possible to observe autosomal UNO-GFP spots in this genotype? If so, what explains the splitting? Is it possible there is a second, weak Separase cleavage site in UNO that is cleaved only slowly (but quickly enough when UNO[nc] levels are diluted)?

-> The revised version includes additional text (results lines 754-769, discussion lines 882-894), a figure (Fig 7E) and a movie (S10 Movie) for a more detailed description of the phenotype resulting from

expression of UNO^{nc}-EGFP in the *uno*+ background. In brief, UNO^{nc}-EGFP dots are present on large autosomes at the start of M I. They get stretched and become weaker when large autosomes separate during exit from M I. However, they are not completely eliminated as in case of UNO-EGFP. Precise quantification is impossible because of the low intensity of the autosomal dots. Further work will be required for a molecular explanation as to why separation of large autosomes succeeds when UNO^{nc}-EGFP is expressed in the *uno*+ background. Presence of additional low efficiency separase cleavage site(s) is not excluded. We discuss this and additional possibilities.

11. Fig. S5 – I believe the authors when they say the images in this figure are illustrative, but there really should be some quantification. This is the only actual evidence in the paper that anaphase II segregation is normal in uno mutants.

-> As suggested, we have further detailed the description of chromosome segregation during M II in *uno* mutants including quantification (lines 398-403). Moreover, for better documentation of chromosome behavior during M II in *uno* mutants, we have also added an additional panel B into the revised S5 Fig. The conclusion that sister centromeres segregate regularly in *uno* mutants during M II is actually not just based on time-lapse imaging data. Our XY FISH analysis of *uno* mutant spermatid nuclei provides independent support for this conclusion. This supporting evidence is now also mentioned in the revised manuscript (lines 404-411).

12. Fig. S7 C & D. The authors should explain how they determine which centromeres are associated with which chromosomes – especially for chromosomes 2 and 3. In addition, the colors of the false-colored centromeres aren't sufficiently distinct in this image

-> The different bivalents were identified as described in Chaurasia et al. (2018). This is indicated now in the revised figure legend. Moreover, we have increased contrast for better visibility of the false-colored centromeres.

Reviewer #2:
Our response to the minor concerns was as follows.

1) The most significant criticism is that the data in a few locations lacks quantitation. For example, in Figure 2B, it is difficult to appreciate how often uno mutants have defects in S5 and promet I. The spermatid images look dramatic but how often do they occur? The prometa I images do not look as bad in the mutant. Can the author conclude the defects get progressively worse? And that Uno is not necessary for pairing? Reporting the numbers of chromosomes at each stage would be helpful.

-> We have changed the text (results lines 329-342) for an improved description of the *uno* mutant phenotype. It is now stated that the early stages (S1 – S3) always displayed a normal pattern of DNA staining and that S5/6 were usually and prometaphase I almost invariably abnormal.Moreover, in postmeiotic cysts, size and DNA content of the spermatid nuclei was always far less homogenous compared to controls.

Unfortunately, chromosome territories have irregular shapes with vague borders during S3-S5. Even in wild type, a large autosomal territory has an appearance that is bi-lobed to varying degrees during S5. It seems impossible to define reliable criteria for scoring territories unequivocally as prematurely separated or not. Without a scientifically robust procedure, we cannot deliver statistical numerical data reporting the number of chromosomes at each stage. Maybe using artificial intelligence in future.

We have not specifically addressed whether *uno* is required for pairing (for example using the lacO-lacI-GFP dot system described by Vazquez et al. 2002). However, the entirely normal appearance of chromosome territories at the S3 stage in *uno* mutants suggests that chromosome pairing is presumably not dependent on *uno* function. Moreover, as also described in our manuscript, some bivalents are clearly still intact at the start of the first meiotic division in *uno* mutants. This finding also indicates that pairing is not strictly dependent on *uno* function.

2) Figure 2F is hard to interpret without a wild-type image. The univalent is obvious, as is the aberrant segregation at anaphase, but it is unclear if the other chromosomes are misbehaving in Prometa I. How rapidly do the bivalents dissociate?

-> For comparison, we have added a wild-type image to Fig 2F.

3) What is the sample size in 2E and 2G? It is also a little surprising there is no genetic measures of nondisjunction.

-> Sample sizes were given in the original S4 table. In the revised manuscript, they are now also given in the figure legends (lines 309, 325).

4) Figure 3 could use more single channel images, especially for UNO in panel C. Can the authors also confirm whether the autosomal foci are never seen with antibody? (suggested in line 361-363). Can the authors give an estimate of how often the autosomal signals are observed?

-> As suggested, we have added UNO grey value images to Fig 3C.

Concerning the sensitivity of anti-UNO for detection of autosomal signals: In our hands, immunofluorescent stainings always have more non-specific fine background specs in comparison to

direct GFP fluorescence detection. After expression of EGFP fusions of UNO, MNM or SNM, we observe weak GFP dots only in autosomal territories but not elsewhere in the cell. Moreover, these signals are lost in *tef* mutants, suggesting that they are specific. Staining of *uno* mutants with anti-UNO results in some randomly distributed non-specific fine background specs that can be just as intense as the potentially specific anti-UNO signals in autosomal territories of *uno*+ spermatocytes. After immunolabeling spermatocytes expressing SNM-EGFP (or MNM-EGFP) with anti-UNO and red fluorescent secondary antibodies, we find anti-UNO signals in weak dots on autosomal territories that are colocalized with weak SNM-EGFP (or MNM-EGFP) dots. Therefore, UNO in autosomal dots can be detected with anti-UNO. However, without marking autosomal AHC dots with some fluorescent AHC protein, anti-UNO signals on autosomes are not necessarily distinguishable from non-specific background specs.

5) In Line 201: can the authors provide any more information on why they picked CG8712 for detailed analysis?

-> Beyond being among the strongest interactors, the selection of CG8712 for detailed further analysis was also motivated by its preferential expression in testis according to FlyBase transcriptome data. We have added this into the revised version (line 228).

6) In Figure 4, it seems that UNO most strongly affects MNM, and SNM most strongly affects UNO. Therefore, can the authors conclude any sort of hierarchy, such as SNM – UNO – MNM? (MNM depends on UNO and UNO depends on SNM). Granted, it is not clean and there are partial dependencies between all members as expected for a complex. It is just surprising that if it is a complex, that UNO localizes quite strongly in a mnm mutant.

-> The proteins MNM, SNM and UNO are clearly interdependent with regard to dot localization on the XY bivalent during prometaphase I. However, the dependencies of these proteins on each other with regard to protein stability and diffuse nucleolar localization are somewhat distinct for different pairs. While speculations on possible meaning of these differences might be possible, we prefer to wait for future results from more detailed analyses.

7) Is the strong UNO on the X affected by tef? It looks that way in Fig 4C.

-> Further detailed signal quantification will be required to resolve whether UNO levels on the XY bivalent might be somewhat reduced.

8) It would help to have single channel images of UNO in Figure 4C, particularly for the images showing the single nucleus and lack of autosomal signals (as is done for mnm-). This should include a wild-type single nucleus image to compare to the mutants in Fig 4C.

-> As suggested, we have add a grey value channel in Fig 4C. We have also added a grey value channel in Fig 3C. As a result, there are now directly comparable figures for single S5 nuclei in wild-type (Fig 3C), *mnm* and *tef* mutant (Fig 4C).

9) In Figure 4C, the UNO signal on the X-Y pair looks dimmer in the tef mutant.

-> see point 7) above

10) Why was the cleavage assay was done in S2 cells. Does it not work in testis?

-> We have made several attempts using various transgenes and drivers to express the separase cleavage reporters for analyses during the meiotic divisions. However, so far we have not been successful with expressing reporters at a level compatible with analysis. Not just insufficient but also excess levels of expression preclude the visualization of centromeric dot signals. Technically, it has been easier to control the level of expression in cultured cells.

11) Figure 7D shows segregating autosomes with a slight delay. How often is this observed? This panel is presented but there is no data on how often this was repeated or the delays observed. When the autosomes segregate, do they have autosomal UNO?

-> As explained above (reviewer 1, point 10), we have extended the description of the effects of UNO^{nc}-EGFP in the *uno*+ background.

12) Do other AHC proteins have separase cleavage sites?

-> MNM and SNM have many matches to the minimal consensus of separase cleavage sites ([E/D]XXR), but none of them has the same strong conservation within drosophilids as the functional site in UNO. In general, the very large majority of matches to the consensus are false positives; they are not true separase cleavage sites. We have generated separase cleavage reporter proteins including full length MNM, SNM, or TEF, without obtaining evidence for cleavage by separase. The revised discussion includes corresponding comments (lines 829-832).

13) The paragraph starting in line 619 appears to contradict itself. The first line suggests that there is a relationship between UNO and kleisins. Line 630 suggest the data argues against a relationship. What do the authors favor? The idea that UNO has at least a kleisin-like role is attractive given the results of this paper. This is particularly important and suggests that, despite the lack of SMC1 and SMC3, that SNM/MNM/UNO form a ring complex that holds homologs together. Or can the authors suggest another mechanism for how Separase cleavage allows the homologs to separate?

-> We have revised the part of the discussion on relatedness of UNO with α-kleisins (lines 827-860). Overall, the protein sequences of UNO, SNM and MNM are so distinct from those of α-kleisins, SMC1 and SMC3 that assumption of a ring complex (formed by UNO, SNM and MNM) homologous to cohesin cannot be based on any support. Whether AHC proteins nevertheless form a ring complex (not homologous to cohesin) is clearly an interesting issue, but its resolution will require far more experimental work.

Reviewer #3:
Our response to the minor comments was as follows:

1) Can the authors show a control in Figure 2F? For somebody not working on drosophila this would be helpful.

-> As suggested, we have included a control in Fig 2F.

2) Figure 4C: It would be nice if the authors could show all stages (prometaphase I and S5) for all genetic backgrounds and the control

-> The revised Fig 4C contains additional single channel images, making comparison to control images shown in other figures straightforward.

3) Concerning the Separase cleavage site I was wondering whether the authors have been able to identify a Separase docking site downstream of the cleavage site (LPE, Rosen et al. Nat Comm 2019). Are there Separase docking sites in other Separase substrates in drosophila?

-> As now also indicated in the revised discussion (lines 837-838), we have not found docking sites within UNO and MNM, while SNM includes one match.
