# Supplementary material for: Chromosome separation during Drosophila male meiosis I requires separase-mediated cleavage of the homolog conjunction protein UNO
Source: PLoS Genet. 2020 Oct 1;16(10):e1008928. doi: 10.1371/journal.pgen.1008928 (PMC7529252; doi:10.1371/journal.pgen.1008928)
Supplement: Attachment — Submitted filename: Review of Lehner ms_mckee.docx [file pgen.1008928.s023.docx]

Review of Weber et al – Bruce McKee

This manuscript reports the discovery, by a co-IP/mass spec procedure from mass-purified Drosophila testes, of a new protein component of the alternative conjunction complex (AHC), which conjoins achiasmate homologs in Drosophila male meiosis and enables their regular segregation. This complex, previously known to include meiosis-specific proteins MNM and SNM (which served as baits in the authors’ procedure), is important both because it is the only well-characterized alternative to chiasmata in connecting meiosis I homologs and because of its as-yet mysterious ability to specifically pair homologs. In addition to its discovery, the authors report a thorough functional characterization of UNO, revealing it to be a card-carrying conjunction protein with all the same phenotypic and chromosome localization properties and co-dependencies as SNM and MNM. The authors use live and fixed imaging of testes to show convincingly that loss of UNO, like loss of MNM or SNM, abolishes conjunction and leads to random segregation of homologs. Taken together with their evidence for strong interactions between UNO and both SNM and MNM, these data confirm the longstanding hypothesis that the AHC really is a protein complex. The authors go on to show, by a beautiful series of experiments, that UNO has one key property SNM and MNM don’t have – a functional Separase cleavage site that provides the means by which Separase activity at anaphase I can release the AHC and liberate homologs. This finding thus answers a crucial question about homolog segregation, one posed a few years ago by the surprising observation (by the same lab) that Separase is required for homolog segregation in Drosophila despite strong evidence that cohesins (and thus alpha-kleisins, the known targets of Separase in chromosome segregation) do not appear to contribute to AHC function. The authors’ data, which include results both of a bio-sensor assay in cultured cells and an in vivo analysis based on site-directed mutagenesis of the cleavage site are fully convincing and thus identify a new Separase target. This is a rare accomplishment in itself. Unfortunately UNO proved not to have any other informative biochemical properties or protein domains, so does not further our understanding of how the AHC pairs homologs. But the authors have provided the meiosis community with a lengthy list of other proteins that co-purify with SNM and MNM as well as a procedure by which testes can be isolated in mass quantities for biochemical and omic studies without the tedium of hand-dissection. This is an important study that will be of broad interest to the meiosis and chromosome communities (and to Separase specialists). The manuscript is well written and devoid of unsubstantiated claims. I have no major criticisms. I have a few minor criticisms related to how the data are presented, detailed below, attention to which would strengthen an already excellent manuscript.

- The authors’ method for purifying testes expressing MNM-GFP or SNM-GFP seems to have worked at least fairly well since they found UNO. But the same argument does not hold for the eGFP control, making it more difficult to assess what value the control provided. Moreover, beyond UNO, the value of the list of interactors in Table S1 to the community at large depends on how pure the testis preparations were, which is not mentioned. In addition to final purity, it would be helpful to investigators who wish to try this method to provide information about how well the major steps in the testis purification protocol worked. It would also be helpful to detail, if possible, whether and to what degree the proteins recovered exhibit a bias toward testis-specificity or enrichment and a bias against specific expression in other organs.
- Line 1197 & Fig 2B Contrary to the statement, I cannot see 8 univalents in PMI in the RNAi cell although I can see them in the uno- mutant.
- Fig. 2D & E It is very hard to distinguish the red+green dots from the green and red ones in the mutant. It would help greatly to present side-by-side images of the separate fluorescence tracks and a merge image without the DNA. This figure is important because it is the only actual evidence for the claim that the X and Y chromosomes segregate randomly. The quantification is also incomplete in that it does not show the actual numbers of each class of nuclei. The authors’ hypothesis predicts not only an equal ratio of regular and nondisjunctional spermatids but also an equal ratio of XY and nullo-XY spermatids. An excess of nullos could indicate that there is some meiosis II nondisjunction as well.
- Relative to the issue of how the X and Y segregate, the most direct approach would be to score anaphase I and telophase I nuclei for distribution of the red and green FISH spots. This isn’t any harder than scoring spermatids and is more direct.
- Fig. 2F is very clear and it is easy to see how the images could be used to assess the overall segregation pattern. But actual numbers of cells scored should be provided for Fig. 2G (and 2C and 2E as well).
- In Figs 2C and 2E the meaning of the error bars needs clarification. I think each cyst is treated as a separate experiment and the error bars represent the differences between the averages for each cyst, but I’m not sure. Also did all the cysts come from one testis or were different males sampled?
- Fig. 3A - At the low magnification shown and in the absence of stage markers, it is hard to tell whether UNO signals are present in spermatogonia. Can the authors provide more definitive evidence to support their claim that UNO is expressed only in spermatocytes?
- Fig. 4B - It is not clear exactly what the arrows in the SNM nucleus are pointing at: UNO or SNM-eGFP or both? Presenting individual images of the separate tracks would clarify.
- Fig. 4C - there appears to be some weak staining of autosomes in the tef- overexposed image. The quantification to the right focuses on spot numbers but much of the staining is diffuse. Is there a way to express the overall difference in staining of autosomes between tef- and tef+?
- Fig. 7 & movie S9 – the ability of the autosomal bivalents in the uno+ background to segregate despite being, presumably, held together by uno[nc] is surprising. The authors point out this interesting behavior but do not offer an explanation. I’m curious whether it is possible to observe autosomal UNO-GFP spots in this genotype? If so, what explains the splitting? Is it possible there is a second, weak Separase cleavage site in UNO that is cleaved only slowly (but quickly enough when UNO[nc] levels are diluted)?
- Fig. S5 – I believe the authors when they say the images in this figure are illustrative, but there really should be some quantification. This is the only actual evidence in the paper that anaphase II segregation is normal in uno mutants.
- Fig. S7 C & D. The authors should explain how they determine which centromeres are associated with which chromosomes – especially for chromosomes 2 and 3. In addition, the colors of the false-colored centromeres aren’t sufficiently distinct in this image.
